# Deoxycytidylate Deaminase and Thymine Catabolic Activity of Transplanted Mouse and Rat Hepatomas and their Histology

**DOI:** 10.1038/bjc.1961.17

**Published:** 1961-03

**Authors:** P. Emmelot, J. F. Hampe, C. J. Bos, I. H. M. Reyers

## Abstract

**Images:**


### DEOXYCYTIDYLATE DEAMINASE AND THYMINE CATABOLIC ACTIVITY OF TRANSPLANTED MOUSE AND RAT HEPATOMAS AND THEIR HISTOLOGY

P. EMMELOT, J. F. HAMPE, C. J. BOS AND I. H. M. REYERS

*From the Departments of Biochemistry and Pathological Anatomy, Antoni van Leeuwenhoek-Huis : The Netherlands Cancer Institute, Amsterdam, The Netherlands.*

Received for publication November 8, 1960

PITOT and Potter (1960) and Potter, Pitot, Ono and Morris (1960) very recently studied various normal tissues and rat hepatomas for the presence of deoxycytidylic acid (dCMP) deaminase (compare also : Maley and Maley, 1959). The enzyme could be demonstrated in embryonic rat liver, in adult liver after induction of bile duct cell proliferation and in a number of the rat hepatomas. Since parenchymal cells of normal adult rat liver did not appear to contain the enzyme, it was considered likely that rat hepatomas which showed dCMP deaminase activity were derived from bile duct or closely related cells (Pitot and Potter, 1960). All rat hepatomas studied by Potter *et al.* (1960), except one, were unable to convert [2–$^{14}$C] thymine to $^{14}CO_2$, but rat liver was markedly active in the latter respect. These findings pointed to the non-uniform metabolic behaviour inherent to individual tumours, a phenomenon frequently encountered in this laboratory among mouse and rat hepatomas and other tumours (Emmelot, Bos, Brombacher and Hampe, 1959).

Experiments directed towards finding a consistent difference between the enzymic profiles of normal and cancer tissues may ultimately help to clarify the cause and the nature of neoplastic growth and provide clues for a rational chemotherapy. The great diversity in results makes it imperative to concentrate on a few tumour types and to study these extensively. Even then, many tumours of a given kind have to be studied in order to obtain a sufficient amount of data for any valid conclusion, in view of the non-uniform enzymic properties of even those tumours which originate from the same cell type, *e.g.* mouse hepatomas (Emmelot *et al.*, 1959 ; Levintow, 1954). Moreover, the question has to be answered first whether or not one has an appropriate normal cell for comparison. This requirement will undoubtedly restrict the choice of the kind of tumours to be investigated. The system mouse-liver-hepatoma appears a suitable one from the point of view of comparability of cell types but a restriction is imposed on the system rat-liver-hepatoma by the fact that the rat hepatoma may be derived from parenchymal and/or bile duct cells.

In the studies of Potter and co-workers a beginning has been made with the investigation of two important problems. First, may the presence of a certain enzyme, such as the dCMP deaminase, in a rat hepatoma serve as a marker for the cell origin of this tumour (Pitot and Potter, 1960 ; Potter *et al.*, 1960) and, secondly, is the deletion of catabolic enzymes of nucleic acid metabolism a consistent feature accompanying neoplastic growth (Potter, 1958, 1960 ; Potter *et al.*, 1960), and if so, is the deletion of qualitative or of quantitative significance ?

In other words, is the deletion of nucleic acid catabolic enzymes a *conditio sine qua non* for cancer or is it promoting cancerous growth? In the latter case some measure of correlation between the particular enzyme profiles and the growth rate of the tumours, or more generally, the stage of tumour progression (Weinhouse, 1960) might exist. Some evidence of such a correlation in regard to thymine catabolism was obtained (Potter *et al.*, 1960).

Since for the elucidation of these highly interesting questions the study of as many hepatomas as possible is called for, Dr. Potter encouraged us to test some of the hepatomas kept in our laboratory for the above enzymes, and kindly provided us with the necessary information before the publication of the papers referred to above. The present paper is concerned with the results of these studies, carried out on three mouse and four rat hepatomas of which other metabolic properties had been (Emmelot *et al.*, 1959) or were being investigated at that time.

### MATERIALS AND METHODS

*Tumours.*—The mouse hepatomas T 28012 and T 26473 were of spontaneous origin and transplanted subcutaneously on (C57 Black × DBA)$F_1$ and ($O_{20}$ × DBA)$F_1$ mice, respectively. Mouse hepatoma CBA 244 had been induced by feeding *o*-aminoazotoluene to a female CBA mouse and was subsequently carried by animals of the same strain. The former two tumours had been transplanted during more than 5 years, the latter tumour was studied in its 11th and 12th transplant generation.

The rat hepatomas had been induced by feeding 4-dimethylaminoazobenzene to female rats of the inbred strain R-Amsterdam, maintained on a polished rice diet. The tumours were transferred intraperitoneally in rats of the same strain. Transplant generations tested for enzyme activity were in the case of hepatoma BY 225 : 98, 101 and 102 ; BY 446 : 11, 13, 17, 20, 22 and 26 ; BY 448 : 9, 10, 11 and 20 ; and BY 463 : 3, 4 and 7. The transplants of hepatomas BY 225, BY 446 and BY 448 were harvested 6–8, 9–12, and 10–12 days after inoculation, respectively. The transplants of hepatoma BY 463 were grown from 3 to 5 weeks, depending on the transplant generation. The hepatomas were studied histologically using conventional techniques (Emmelot *et al.*, 1959) ; hepatoma BY 225 has been described previously (Emmelot *et al.*, 1959).

*Rat hepatoma BY* 446.—The primary hepatoma was mainly of the adeno type ; the possibility might exist that part of this structure was due to lymphatic cysts which caused the surrounding tumour cells to attain an adenomatous appearance. Transplant generation 1 : mixed adeno-solid hepatoma ; transplant generations 2–20 : solid hepatomas containing cysts, the latter gradually disappearing so that a more uniform structure ensued ; transplant generation 20 : exclusively solid hepatoma, a few cysts ; transplant generations 22–26 : as 20, in one transplant of generation 26 more polymorphy and mitoses than in the others ; compare : Fig. 1 to 5.

*Rat hepatoma BY* 448.—The primary hepatoma was of the mixed solid-adeno type, lymph cysts were present. Transplant generation 1 : Only a few adenomatous structures, in part resembling those of the primary tumours. In this and the following transplants the character of the cells lining the tubules gradually gained the appearance of cells of the solid type and resembled less the polymorphic mucous producing cells of the cholangio type ; transplant generations 6–10 : no cysts, uniform solid structure resembling the epithelial type of liver cells ; transplant generations 11–20 : solid hepatomas, a few parts consisting of fusi-form (sarcoid) cells, adenomatous structures only very scarcely present, at some places trabecular arrangement of the cells but the latter was not characteristic for the whole tumour ; compare : Fig. 6–10.

*Rat hepatoma BY* 463.—The primary tumour was the only one studied in the present series which was almost exclusively of the solid type. Some cysts, slight haemopoiesis and hyaline bodies were present. Upon transplantation no change in the overall structure occurred, a slight haemopoietic reaction was observed in the transplants but the hyaline bodies disappeared ; compare : Fig. 11 and 12.

*Enzymic determinations.*—Enzyme activities were studied in 105,000 × g supernatants prepared according to Pitot and Potter (1960) and Potter *et al.* (1960). Deoxycytidylate deaminase activity was determined according to Maley and Maley (1959) and Pitot and Potter (1960). [2–$^{14}$C] Thymine breakdown was studied using the system of Canellakis (1958) as modified by Potter *et al.* (1960). Enzyme activities were expressed in units, one unit standing for 1 $\mu$mole of deoxyuridylic acid formed or [2–$^{14}$C] thymine disappeared/amount of supernatant derived from 1 g. of fresh tissue/60 minutes at 37° C. The data listed in parentheses in Table II have been expressed per mg. of nitrogen of the soluble fraction.

### RESULTS

Table I shows that the dCMP deaminase was completely missing from normal adult mouse liver and from the three transplanted mouse hepatomas selected for the present study. Two of the hepatomas, T 28012 and T 26473, showed negligible

---

### EXPLANATION OF PLATES

(All slides 6 m$\mu$, fixation Susa, stained with haematoxylin azophloxin.)

FIG. 1–5.—The BY 446 rat hepatoma. ×130.

FIG. 1–2.—Primary tumour : main pattern adenomatous, but combined with pseudo-adenomatous structures (lymphatic cysts ?) and solid trabecular parts (especially Fig. 2).

FIG. 3.—First transplant : adenomatous and solid.

FIG. 4.—Twentieth transplant : solid trabecular hepatoma throughout (some lymphocysts). Rather uniform pattern, moderate amount of mitoses, some pleomorphism.

FIG. 5.—Twenty-sixth transplant : solid trabecular hepatoma as in Fig. 4, a few adenomatous structures, conspicuous cyst formation, exhibiting more pronounced pleomorphism and mitotic activity than the other transplants 20–26 (see text—the present transplant contained 51 dCMP deaminase units). Other transplants (20–26) more like the one illustrated in Fig. 4.

FIG. 6–10.—The BY 448 rat hepatoma. ×130.

FIG. 6.—Primary tumour : adenomatous part with inflammatory cells typical of this structure, mucus producing. Some haemopoiesis (not visible in this figure).

FIG. 7, 8.—First transplant : mainly solid with some adenomatous structures and solid trabecular parts forming slits of a regular (quasi-adenomatous) type.

FIG. 9.—Sixth transplant : solid hepatoma, shows slight slit formation lined by cells of the solid hepatoma type. At the right some quasi-adenomatous structures probably due to lining around infiltrated fat tissue.

FIG. 10.—Eleventh transplant : solid hepatoma, some necrosis and fibrosis (" old " transplant), increasing pleomorphism, slight fusicellular arrangement in some parts as illustrated here.

FIG. 11–12.—The BY 463 rat hepatoma.

FIG. 11.—Primary tumour (×130) : solid hepatoma of regular appearance, some cysts.

FIG. 12.—Third transplant (×340) : solid hepatoma with an overall structure resembling that of the primary tumour, slight haemopoiesis.

1

2

3

4

Emmelot, Hampe, Bos and Reyers.

Emmelot, Hampe, Bos and Reyers.

9

10

11

12

Emmelot, Hampe, Bos and Reyers.

activity in converting carbon atom 2 of thymine to carbon dioxide but hepatoma CBA 244 was about half as active as normal mouse liver. Adult rat liver appeared to be about twice as active as mouse liver but all four rat hepatomas studied possessed very little, if any, thymine catabolic activity. By contrast, the latter hepatomas showed various levels of dCMP deaminase activity.

TABLE I.—*Deoxycytidylate Deaminase and Thymine Catabolism in* 105,000 × g *Supernatants from Mouse and Rat Hepatomas*

Enzyme activity in units, as defined under Methods.

| Tissue | Deoxycytidylate deaminase | Thymine catabolism |
|---|---|---|
| Mouse liver . . | 0 . | 1·15 |
| Mouse hepatomas : | | |
| T 28012 . . | 0 . | Insignificant* |
| T 26473 . . | 0 . | 0 |
| CBA 244 . . | 0 . | 0·6 |
| Rat liver . . . | 0 . | 2·3 |
| Rat hepatomas : | | |
| BY 252 . . | 66–88† . | Insignificant* |
| BY 463 . . | 70–77‡ . | ,, |
| BY 446 . . | 8–81§ . | ,, |
| BY 448 . . | 9–33§ . | 0·36 |

* Five or less per cent of added radioactivity, [2- $^{14}$C] thymine, disappearing after 60 minutes, taken as experimental error.

† Transplant generation 102 : 11·2 $\mu$moles deoxyuridylic acid formed/mg. N of soluble fraction/60 minutes.

‡ Transplant generation 7 : 8·0 $\mu$moles deoxyuridylic acid formed/mg. N of soluble fraction/60 minutes.

§ Compare Table II.

It is of interest to compare the latter data with the histology of the hepatomas. The histological classification has been made in purely descriptive terms, the predicates solid (hepatocellular = parenchymous) or adenomatous (cholangio-cellular) have been coined from analogy with the normal situation but do not necessarily refer to the cell origin of the hepatomas as stated in parentheses. This limitation is, of course, inherent to the histological discipline. This being so, it might not be astonishing to find a complete lack of correspondence between the histological classification and the dCMP deaminase levels of the hepatomas if the latter enzyme were to be a reliable marker for the cell origin of the tumours. A correspondence between the two sets of data, however, is by no means to be excluded on *a priori* grounds.

Two of the hepatomas, BY 225 and BY 463, both of an exclusively solid type, possessed high levels of dCMP deaminase activity, like those reported by Pitot and Potter (1960) for the Novikoff hepatoma ; one of these, BY 225, had been transplanted for many generations but the transplants of the BY 463 hepatoma had been carried through a few generations only. The description of these hepatomas as solid apparently does not imply that they were derived from parenchymal cells, since their enzyme levels were at variance with this assumption. Incidentally, neither do these results allow the enzyme levels to be correlated with the number of transplant generations through which the hepatomas had been carried, that is, with their growth rate which may be considered as an aspect of the actual stage of tumour progression.

Hepatomas BY 446 and BY 448, both also of the solid type, showed various levels of dCMP deaminase activity according to the number of generations through which they had been transplanted (Table II). The enzymic activities found in the

TABLE II.—*Deoxycytidylate Deaminase Activity of the Rat Hepatomas BY* 446 *and BY* 448 *in Relation to the Number of Generations through which the Transplants had been carried*

Enzyme activity as μmoles deoxyuridylic acid formed per equivalent of soluble fraction from 1 gram of fresh tissue (or per mg. N of soluble fraction, marked*)/60 minutes.

| Transplant generation | Deoxycytidylate deaminase of hepatoma : BY 446 | BY 448 |
|---|---|---|
| 9 | — | 9 |
| 10 | — | 15 |
| 11 | 8 | 30 |
| 13 | 27 (1·7)* | — |
| 17 | 43 (3·5)* | — |
| 20 | 81 (8·7)* | 33 (2·7)* |
| 22 | 21 (1·6)* | — |
| 26† | 44 (35–51) (4·9)* | — |

† Several transplants of the 26th generation were studied, enzyme units ranging from 35 to 51 (4·1* to 6·0*).

transplants of BY 448 varied from low to moderate, whereas the activities of the BY 446 transplants showed a gradual increase (generations 11 to 20) followed by a decrease (generations 20 to 26). Since one or two transplants of a given generation were used for the enzymic assay, whereas the tumour line was maintained by another transplant of the same generation, an element of variability might have been introduced. The latter, however, does not appear to account for the differences observed in the enzymic activities of the various transplant generations of the BY 446 hepatoma (compare the data on the 26th generation listed in Table II and those mentioned under Materials and Methods). Moreover, such a variability as observed with the BY 446 transplants was not found in the case of the hepatomas BY 225 and BY 463 (Table II), whereas only a moderate increase in enzymic activity was observed with hepatoma BY 448 (Table II). The histological appearance of the various transplants used for the present enzymic determinations did not suggest that changes in the cells populations had occurred in respect to the relative contribution of adeno, dCMP deaminase positive, type of cells.

### DISCUSSION

### *Mouse hepatomas*

The classification of hepatomas as solid and of parenchymal origin is less subject to criticism as to the non-equivalence of these terms in the case of the mouse than in that of the rat. The absence of any dCMP deaminase activity from all three mouse hepatomas studied by us is in accord with what one may expect it to be if the enzyme is confined to bile duct cells.

As regards the thymine catabolizing enzymes, it is of interest that the hepatomas T 26473 and T 28012 take nearly four weeks to reach full size, whereas hepatoma CBA 244 takes five weeks to do so. The finding that the latter hepatoma showed thymine catabolic activity whereas the two former did not, does not, therefore, contradict the concept (Potter, 1958, 1960; Potter *et al.*, 1960) that correlates the deletion of non-essential enzymes with the growth rate of the tumour. It should, however, be pointed out that this correlation does not necessarily indicate that an increased growth rate is the result of the deletion of these enzymes which are non-essential for cell division. The deletion might be part of a more complicated pattern of change, or even constitute merely a secondary symptom accompanying tumour progression, without being in the slightest sense responsible for the increase in malignancy. In this respect it remains of the greatest interest to ascertain to what extent the enzymes concerned with the catabolism of nucleic acid precursors do actually constitute a growth-regulator in normal tissue (Canellakis, Jaffe, Mantsavinos and Krakow, 1959; Hiatt and Bojarski, 1960).

*Rat hepatomas*

The results reported in Tables I and II allow the conclusion that no correlation between the histological data and the dCMP deaminase activities of the rat hepatomas can be established. Obviously, then, at least one of these two means of classification cannot be regarded as a reliable measure for the cell origin of the hepatomas. Either bile duct tumour cells grow in a solid pattern and are adenomatous structures in the hepatoma not equivalent, that is to say related, to similar histological structures in the normal liver, or else the dCMP deaminase activity is not an exclusive property of bile duct and tumour cells derived therefrom, while the enzyme might also be subject to adaptive or mutational events. Since the histological aspect can only be presented in descriptive terms which cannot be translated into direct relationships between the (normal and cancer) cells, whereas the enzymic data do reflect the functional aspects of the cell types, the latter are to be preferred as a criterion for the cell origin of the hepatomas, provided that certain conditions are satisfied. In the remaining part of this discussion we will be concerned with the latter question which, in our opinion, cannot as yet be answered in a positive sense.

The evidence that the dCMP deaminase is a genuine property of bile duct cells is only indirect and stems from the finding that feeding with a carcinogenic azo dye leads to positive dCMP deaminase activity in the liver (Pitot and Potter, 1960). However, apart from the induction of bile duct cell proliferation, the latter treatment also affects the parenchymal cells of the liver (Chang, Spain and Griffin, 1958; Porter and Bruni, 1959).

The finding of Pitot and Potter (1960) that embryonic liver does possess dCMP deaminase activity in contrast to the fully differentiated liver, consisting mainly of mature parenchymal cells, does not necessarily indicate that in the latter case the genetic information for the enzyme has been lost from the parenchymal cells. If only its cytoplasmic expression were to be suppressed as a result of the particular feed-back mechanisms operating in the normal adult liver, the potential information might be released again on the initiation of a new cytoplasmic state of metabolism accompanying the " mutational " conversion of a parenchymal cell to a tumour cell.

Such a phenomenon of physiological adaptation (or " modulation " at the enzymic level) might also come into operation at some time during the natural history of liver tumour cells, either of parenchymal (enzyme : absent → present) or of bile duct cell (present → absent) origin, through an adaptation to progression.

A similar situation would exist if hepatoma cells were to be derived from the cells of a (hypothetical) stem layer in the adult liver, possessing the ability to differentiate to either one of the two cell types, and if such tumour cells retained at least partly, the latter capacity, while the actual expression of the specific properties might be subject to " modulation ". Thus, changes, perhaps of a partly reversible nature, might occur in the enzymology (and morphology) of liver tumour cells which one diagnoses arbitrarily as being either of parenchymal or of bile duct cell origin.

However, if the latter views are dismissed and the dCMP deaminase activity is regarded as a permanent property of bile duct, and not of parenchymal cells, and of the tumour cells derived therefrom, the absence of the enzyme in a hepatoma may still be due to a random mutation by loss in a bile duct tumour cell followed by selection. Changes in activity might be due to partial selection of new or pre-existing variants.

Since the specific activity of the enzyme per normal or cancerous bile duct cell is not known, positive dCMP deaminase activity in a hepatoma leaves one in doubt as to the presence of a mixed population of cells. Some tumour cells of bile duct origin with a " high " specific activity or many such cells with a " low " specific activity may be present, and this might vary from one hepatoma to another.

In the absence of further evidence, positive dCMP deaminase activity in a hepatoma, therefore, merely indicates that an unknown number of tumour cells of bile duct origin (if not of a *pseudo* bile duct type due to " modulation ") are present among a population of other tumour cells which are of parenchymal origin or of bile duct origin after having " lost " the enzyme. Even this conclusion may be complicated by the unknown dCMP deaminase activity of the stroma of the tumour. Histochemical methods may help to solve some of these problems. Such studies are being carried out at present (Willighagen and Planteydt, 1959), and it is hoped to publish the results in forthcoming papers together with electron microscopical observations and further biochemical features of the four rat hepatomas.

### SUMMARY

Enzyme assays for deoxycytidylate deaminase and thymine catabolism were carried out on the soluble fractions from three transplanted mouse and four transplanted rat hepatomas. None of the mouse hepatomas, nor mouse liver, contained any deoxycytidylate deaminase activity but one of the mouse hepatomas, possessing a somewhat slower growth rate than the two others, was about half as active as normal mouse liver in converting carbon atom 2 of thymine to $CO_2$. Normal rat liver was twice as active as normal mouse liver in the latter respect, but the rat hepatomas showed very little, if any, activity. Thymine catabolism in relation to tumour progression is briefly discussed.

The four rat hepatomas carried moderate to high levels of deoxycytidylate deaminase activity. With one hepatoma marked variations were noted according to the number of generations through which the transplants had been carried. No correlation between the histology of the four hepatomas, which were predominantly of the solid type, and enzyme levels could be established. Though, generally speaking, a functional classification of the rat hepatomas based on enzyme profiles should be preferred to a histological one which can be presented only in descriptive terms and does not necessarily refer to the cell origin of the hepatomas, the former procedure is beset with many difficulties. The latter are discussed and the conclusion is drawn that the deoxycytidylate deaminase cannot, as yet, be considered as a proper parameter of the cell origin of rat hepatomas.

### REFERENCES

CANELLAKIS, E. S.—(1958) *J. biol. Chem.*, **221**, 315.
*Idem*, JAFFE, J. J., MANTSAVINOS, R., AND KRAKOW, J. S.—(1959) *Ibid.*, **224**, 2096.
CHANG, J. P., SPAIN, J. D., AND GRIFFIN, A. C.—(1958) *Cancer Res.*, **18**, 670.
EMMELOT, P., BOS, C. J., BROMBACHER, P. J. AND HAMPE, J. F.—(1959) *Ibid.*, **13**, 348.
HIATT, H. H. AND BOJARSKI, T. B.—(1960) *Biochem. biophys. res. Comm.*, **2**, 35.
LEVINTOW, L.—(1954) *J. nat. Cancer Inst.*, **15**, 347.
MALEY, G. F. AND MALEY, F.—(1959) *J. biol. Chem.*, **234**, 2975.
PITOT, H. C. AND POTTER, V. R.—(1960) *Biochim. biophys. Acta*, **40**, 537.
PORTER, K. R. AND BRUNI, C.—(1959) *Cancer Res.*, **19**, 997.
POTTER, V. R.—(1958) *Fed. Proc.*, **17**, 691.—(1960) *Acta Un. int. Cancr.*, **16**, 27.
*Idem*, PITOT, H. C., ONO, T. AND MORRIS, H. P.—(1960) *Cancer Res.*, **20**, 1255.
WEINHOUSE, S.—(1960) *in* 'Amino Acids, Proteins and Cancer Biochemistry' ed. Edsall, J. T. New York (Academic Press Inc.) p. 109.
WILLIGHAGEN, R. G. J. AND PLANTEYDT, H. T.—(1959) *Nature, Lond.*, **183**, 263.


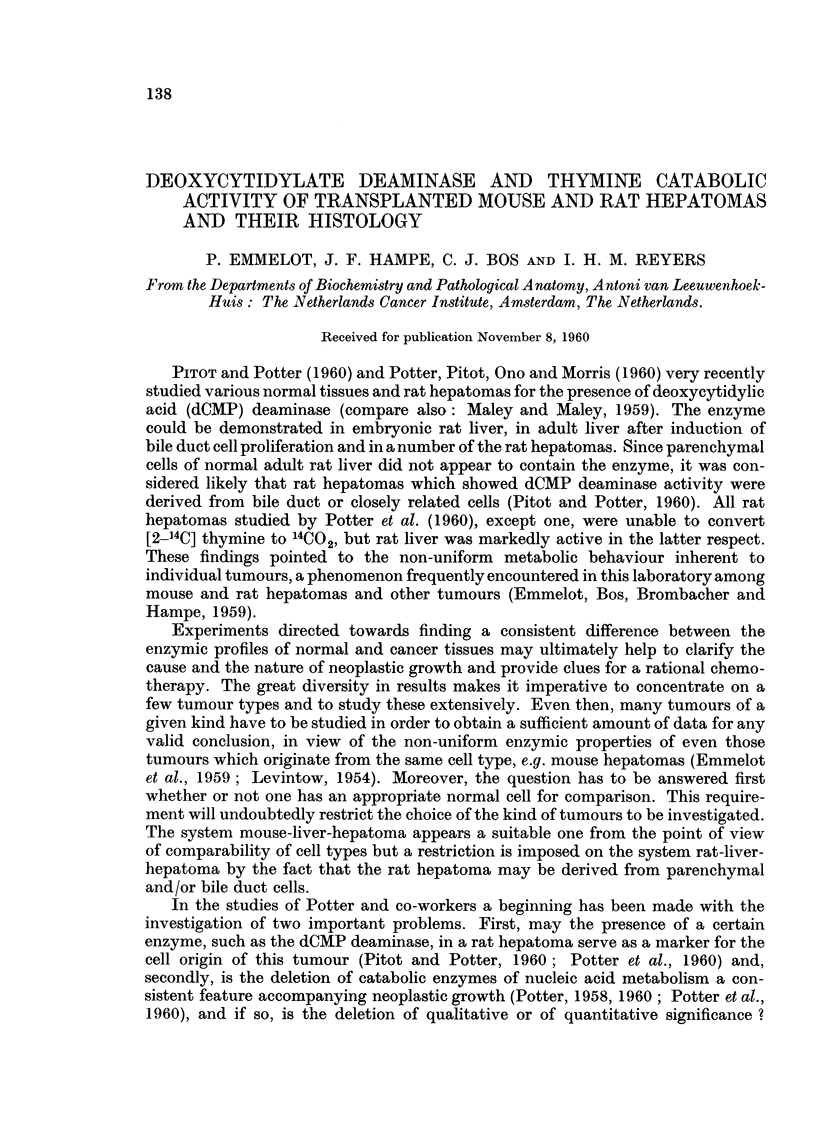

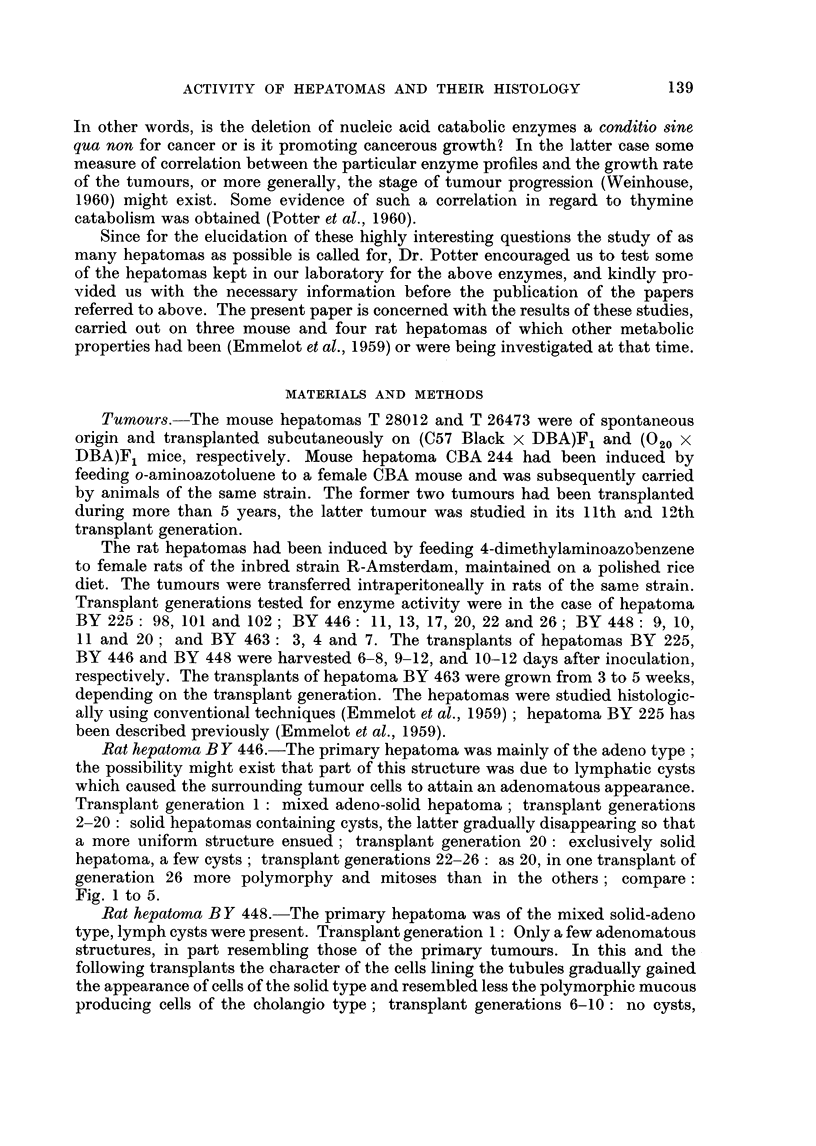

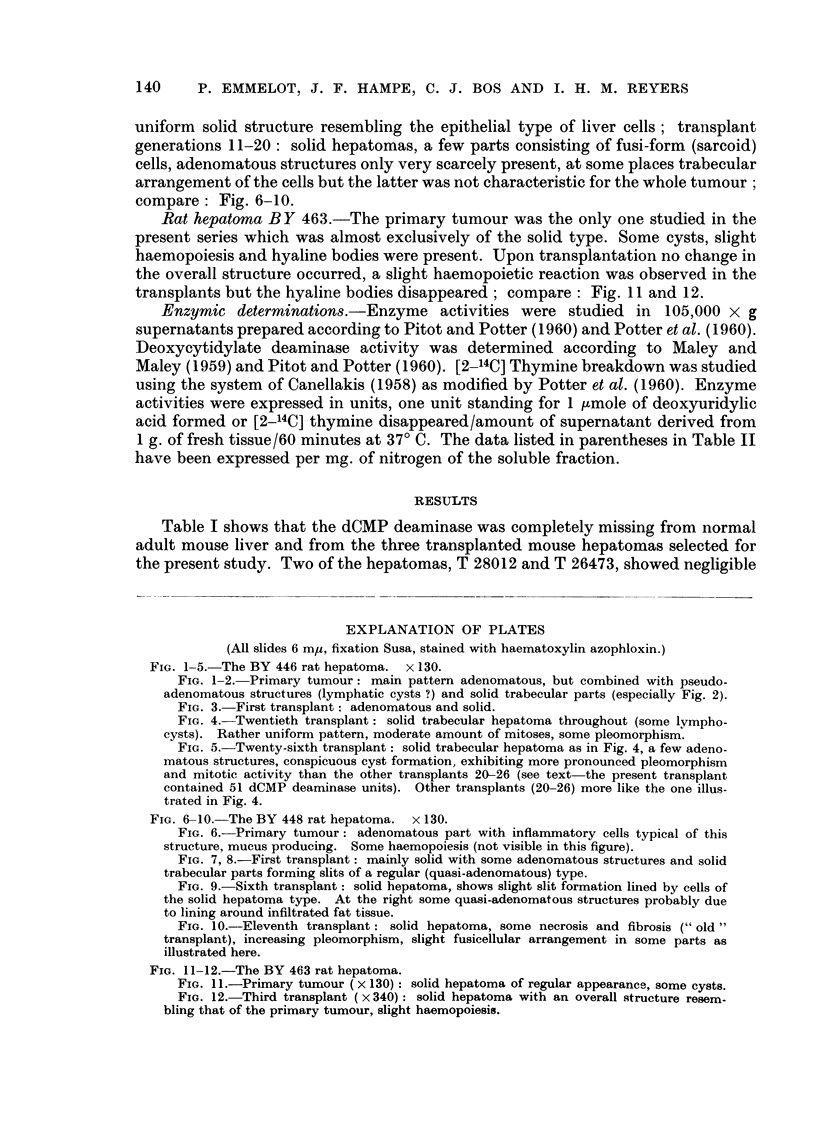

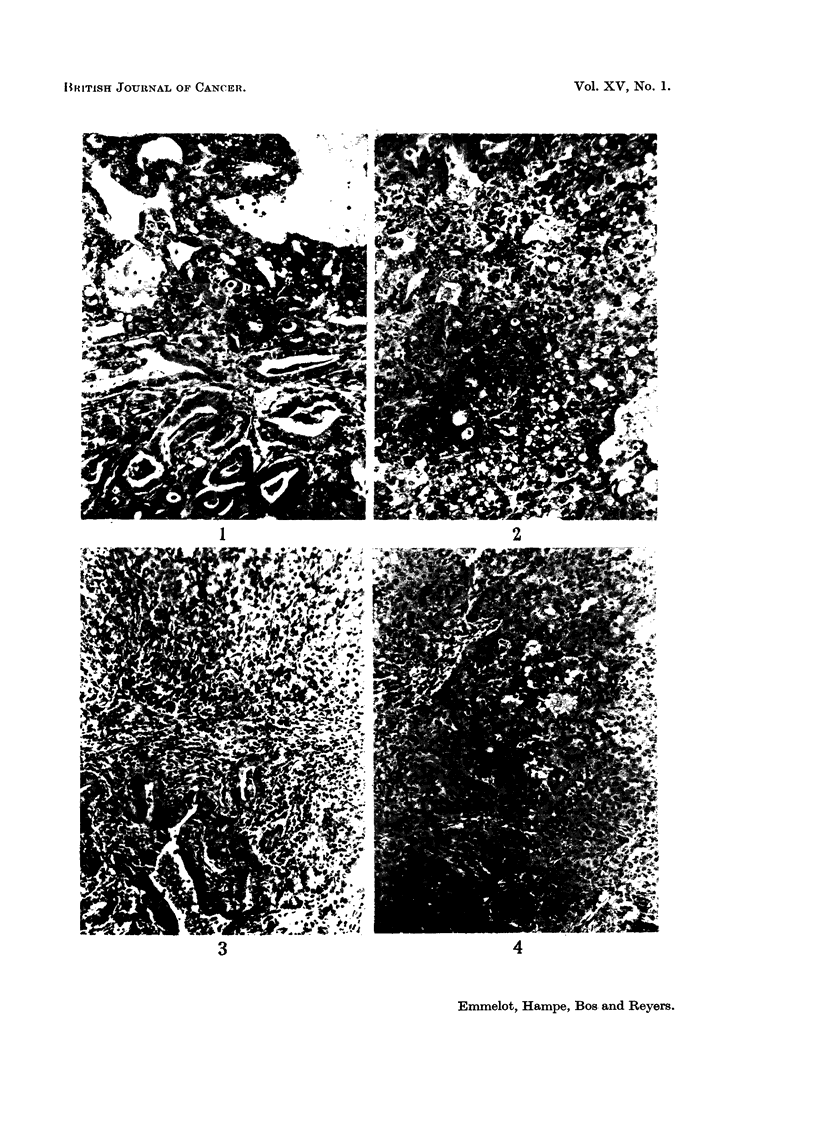

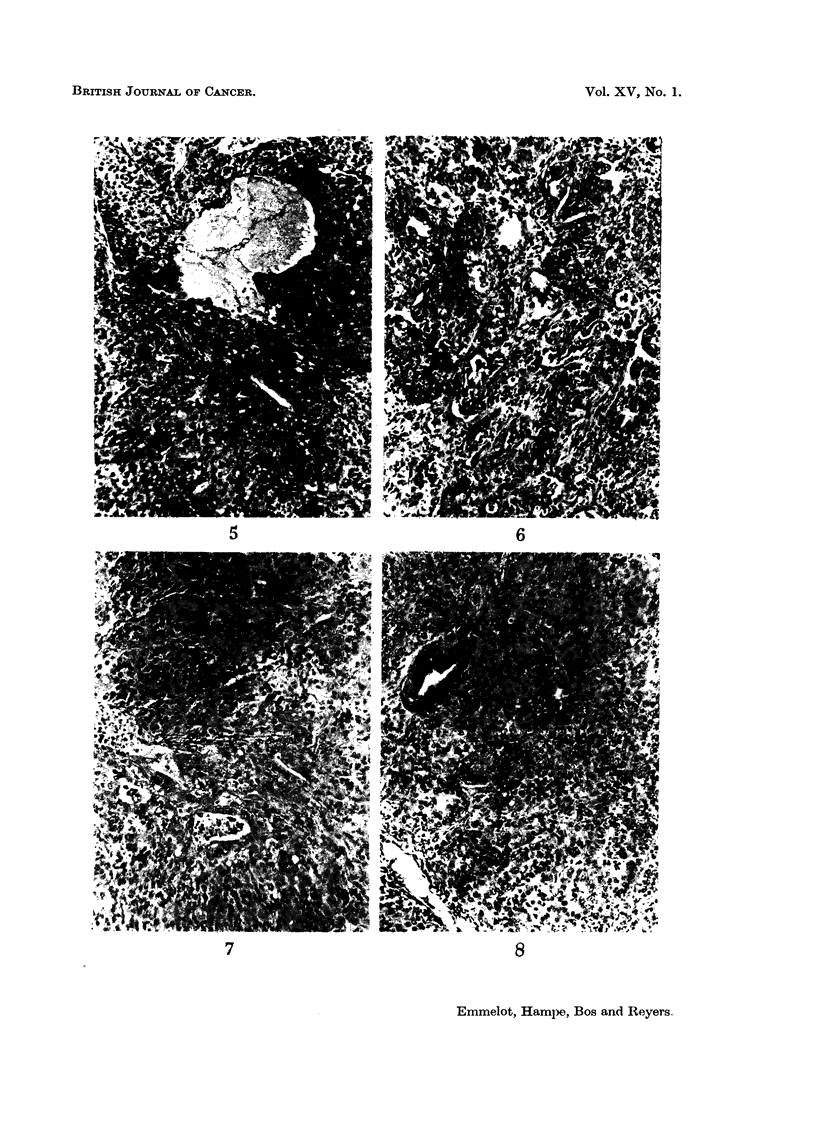

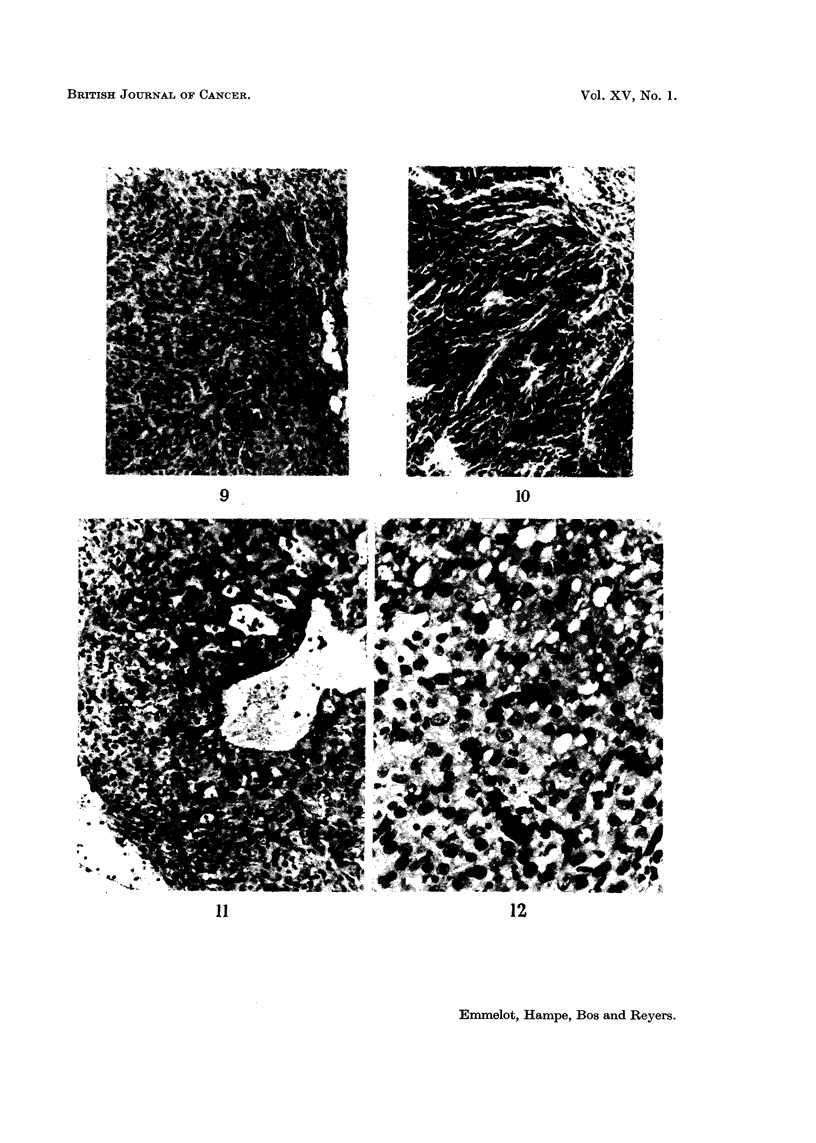

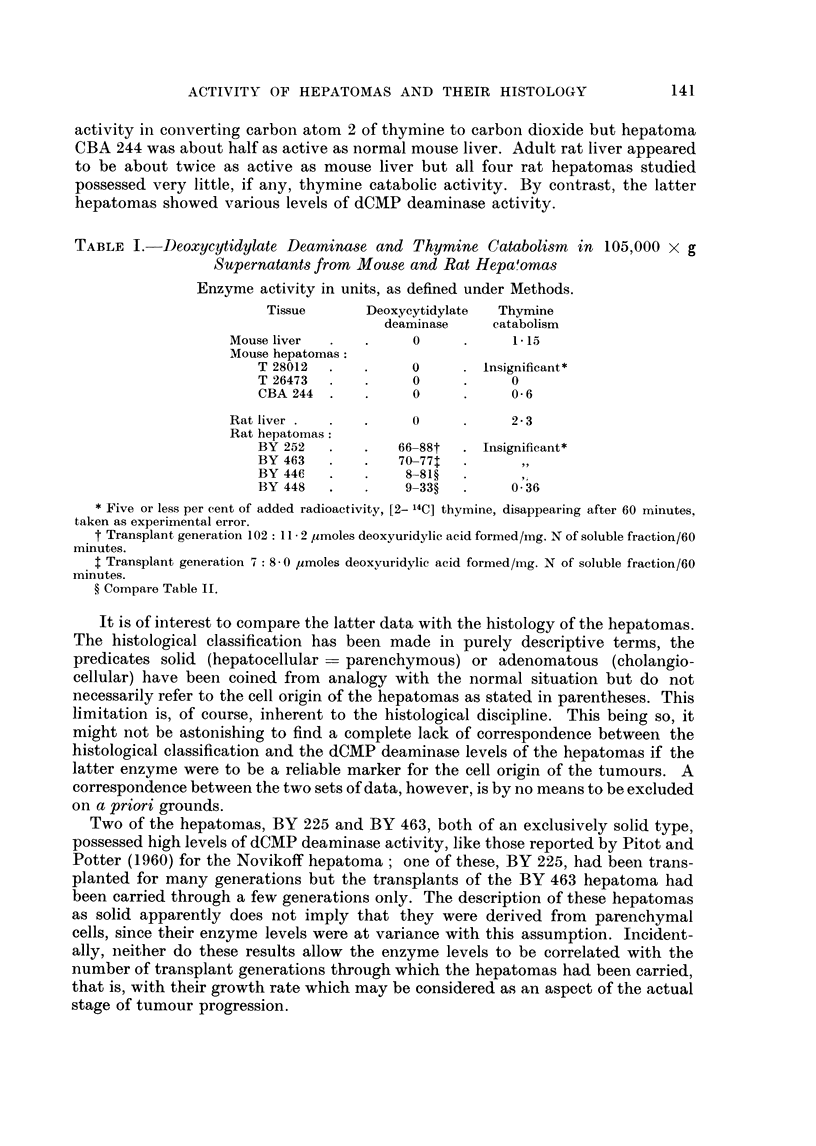

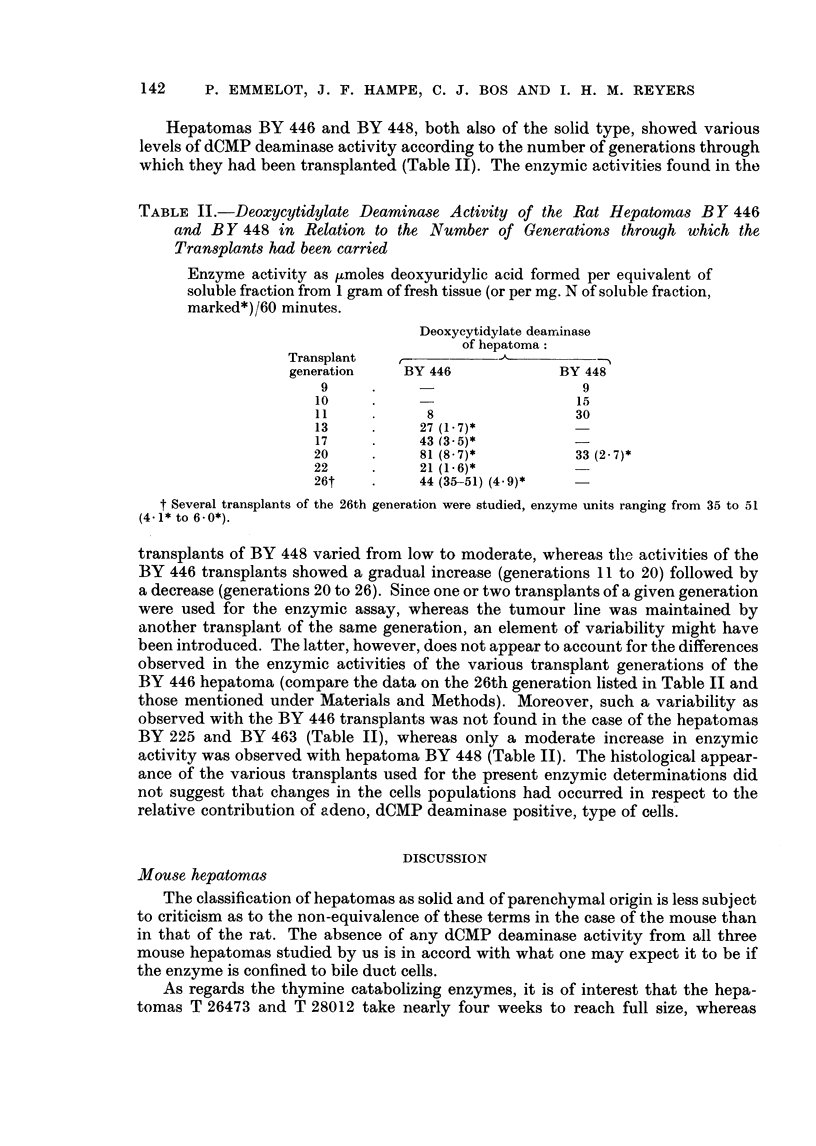

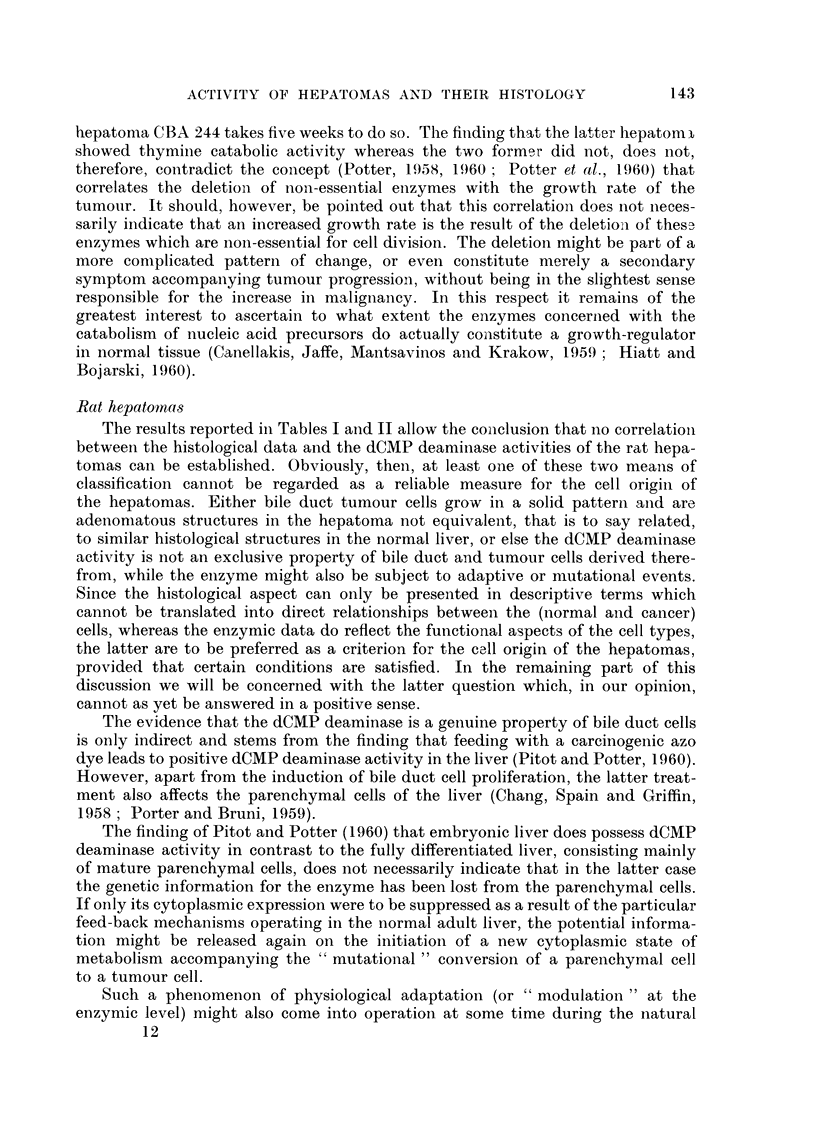

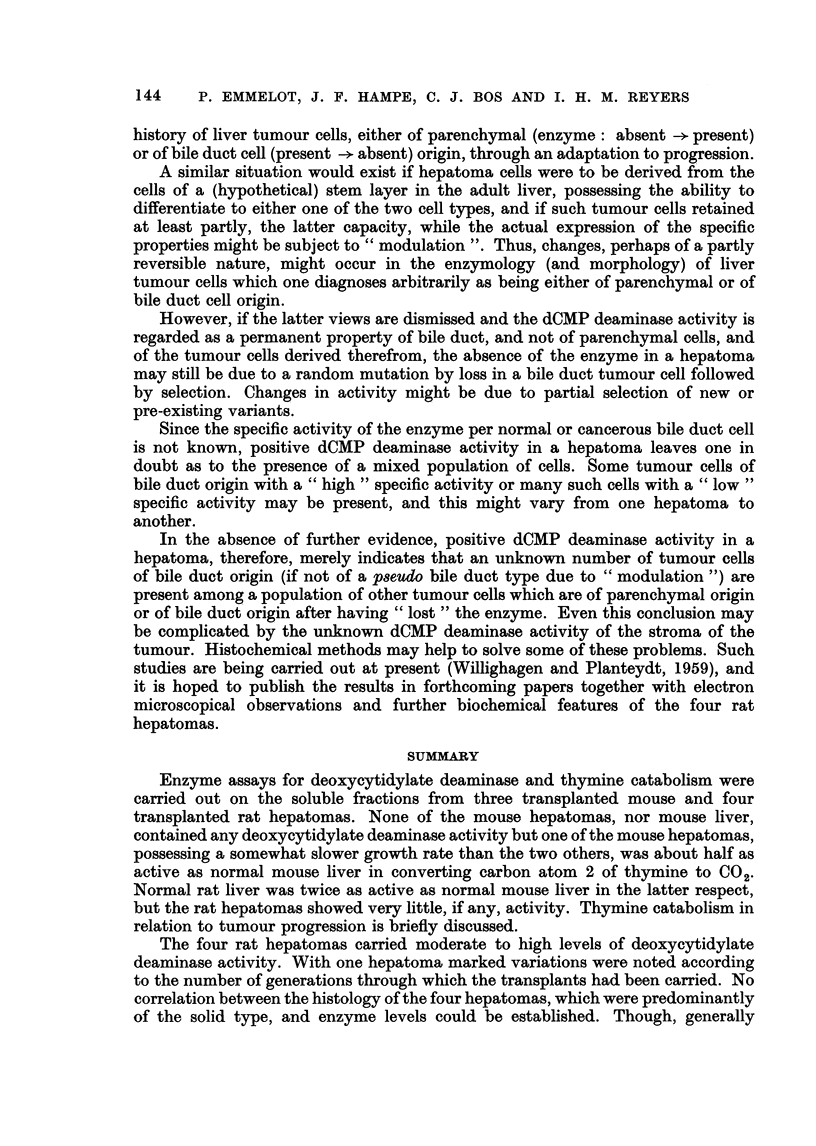

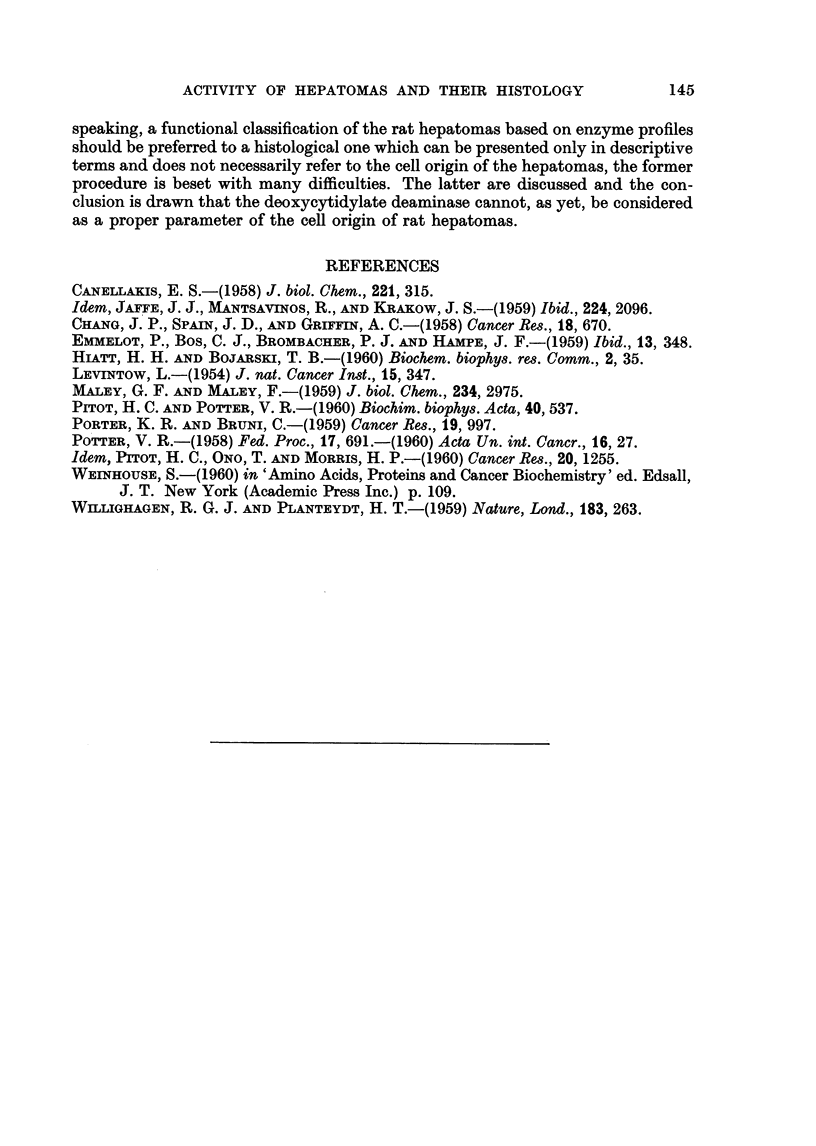


## References

[OCR_00516] CHANG J. P., SPAIN J. D., GRIFFIN A. C. (1958). Histochemical manifestations of early changes in rat liver during carcinogenesis induced by 3'-methyl-4-dimethylaminoazobenzene.. Cancer Res.

[OCR_00518] EMMELOT P., BOS C. J., BROMBACHER P. J., HAMPE J. F. (1959). Studies on isolated tumour mitochondria: biochemical properties of mitochondria from hepatomas with special reference to a transplanted rat hepatoma of the solid type.. Br J Cancer.

[OCR_00520] LEVINTOW L. (1954). The glutamyltransferase activity of normal and neoplastic tissues.. J Natl Cancer Inst.

[OCR_00522] MALEY G. F., MALEY F. (1959). Nucleotide interconversions in embryonic and neoplastic tissues. I. The conversion of deoxycytidylic acid to deoxyuridylic acid and thymidylic acid.. J Biol Chem.

[OCR_00524] PITOT H. C., POTTER V. R. (1960). An enzymic study on the cellular origin of the Dunning and the Novikoff hepatomas in the rat.. Biochim Biophys Acta.

[OCR_00525] PORTER K. R., BRUNI C. (1959). An electron microscope study of the early effects of 3'-Me-DAB on rat liver cells.. Cancer Res.

[OCR_00528] POTTER V. R., PITOT H. C., ONO T., MORRIS H. P. (1960). The comparative enzymology and cell origin of rat hepatomas. I. Deoxycytidylate deaminase and thymine degradation.. Cancer Res.

[OCR_00534] WILLIGHAGEN R. G., PLANTEYDT H. T. (1959). Aminopeptidase activity in cancer cells.. Nature.

